# Ambidextrous Leadership and Presenteeism Among Clinical Nurses: A Moderated Mediation Model of Self‐Efficacy and Leader–Member Exchange

**DOI:** 10.1155/jonm/5209573

**Published:** 2026-10-28

**Authors:** Xiuran Zhou, Shifang Zou, Hui Peng, Ying Yu, Xiaoying Yan

**Affiliations:** ^1^ School of Nursing Guangdong Pharmaceutical University Guangzhou Guangdong China gdpu.edu.cn; ^2^ Guangzhou Municipal Health Commission Guangzhou Guangdong China

**Keywords:** ambidextrous leadership, leader–member exchange, mediating moderating analysis, nurses’presenteeism, self-efficacy

## Abstract

**Aim:**

This study aimed to examine the pathways linking ambidextrous leadership (AL) to nurses’ presenteeism, with a particular focus on the mediating role of self‐efficacy and the moderating role of leader–member exchange (LMX) in this process.

**Background:**

Presenteeism among clinical nurses remains persistently high and is associated with reduced individual productivity and with risks to patient safety. Previous research has examined associations between the leadership style and attendance behavior; however, AL—the flexible combination of exploratory and exploitative behaviors—has received limited empirical attention in this context. Examining this pathway may inform nursing management practice.

**Methods:**

A cross‐sectional, descriptive, and correlational design was adopted. Data were collected from 394 clinical nurses recruited through convenience sampling at three tertiary hospitals in Guangzhou. In addition to correlational analyses, regression models were constructed to examine the mediating and moderating pathways among these variables.

**Results:**

AL was negatively associated with nurses’ presenteeism and positively associated with self‐efficacy; higher self‐efficacy was, in turn, associated with lower presenteeism. Mediation analysis indicated that self‐efficacy partially accounted for the association between AL and presenteeism, with the indirect pathway representing 33.3% of the total effect. In addition, LMX moderated the first stage of this pathway: the positive association between AL and self‐efficacy was stronger among nurses reporting higher LMX.

**Conclusion:**

The findings support the hypothesized moderated mediation model, in which self‐efficacy partially mediates the association between AL and nurses’ presenteeism, and in which LMX conditions the first stage of that pathway. Because the design was cross‐sectional, these associations are correlational and cannot establish causal direction. The findings suggest that structural measures such as reasonable scheduling, sick‐leave protection, workload adjustment, and occupational health support remain the primary responsibility of nursing management and that managerial attention to relational quality and to nurses’ self‐efficacy beliefs may complement, but cannot replace, them.

## 1. Introduction

Presenteeism describes a widely documented workplace phenomenon: employees show up for duty despite being physically or mentally unwell, yet function at far less than their full capacity [1]. As a widespread occupational psychosocial phenomenon, it is associated not only with poorer individual health but also with reduced productivity and, over time, with substantial economic costs [2].

Among all occupational groups, nurses consistently rank near the top of the presenteeism list. A meta‐analysis covering 14 countries estimated the overall prevalence among nurses at 49.2% [2, 3], and estimates from China are similarly high—rates exceeding 50% have been reported in emergency, pediatric, and intensive care units [4–6]. When nurses work through illness, the consequences extend beyond the individual. Their own recovery may be prolonged and, more critically, diminished concentration and delayed responsiveness [7] may compromise the quality of patient care and introduce avoidable safety risks. Reducing presenteeism among nurses, therefore, has become an urgent priority for healthcare administrators.

It is essential to state at the outset that presenteeism is fundamentally an organizational and occupational‐health problem rather than a deficiency of individual nurses. When nurses work while unwell, the primary responsibility of nursing management lies in structural and institutional provision: reasonable and predictable scheduling, adequate sick‐leave protection, workload adjustment, sufficient staffing, and access to occupational health services. These measures protect nurses’ rights and well‐being, safeguard patient safety, and are the foundation of a healthy professional environment. The present study does not contend that nurses should become more psychologically capable of working while ill, nor does it propose that individual‐level intervention can substitute for organizational accountability. Our aim is narrower: to examine one proximal, modifiable mechanism—the relational and psychological pathway—that may operate alongside, and only alongside, adequate structural support. We return to the ethical boundaries of this position in Section 3.2.4 and to its managerial implications in Section 3.2.5.

Prior research has identified leadership style as a meaningful predictor of presenteeism [8]. Leaders shape employees’ on‐the‐job behaviors through two channels: reinforcing work motivation on one hand and reshaping the organizational environment on the other. Yet most existing studies have confined their lens to a single leadership style, which, to some extent, misses the complexity of real‐world management, in which experienced leaders seldom rely on a single fixed approach but instead adjust their behavior flexibly as situations demand.

According to social information processing theory, individuals interpret and integrate cues from their social environment to shape attitudes and guide actions [9]. In the clinical setting, leaders represent the most salient source of such social information for nurses. We, therefore, place ambidextrous leadership (AL) at the center of our inquiry.

AL is not a mechanical blend of transformational and transactional styles but rather emphasizes their dynamic interplay and mutual reinforcement, as originally conceptualized by Rosing et al. [10]. Transformational leadership is theorized to support intrinsic motivation and innovative drive, while transactional leadership is associated with procedural rigor and patient safety through clear goal setting and accountability [11]. In the nursing context, where routine tasks demand high standardization yet emergencies call for on‐the‐spot judgment, neither style alone suffices. Transformational leadership alone may invite procedural lapses; transactional leadership alone may stifle engagement. Their combined use may help address the dual demands of “regulatory compliance” and “adaptive flexibility” and may thereby be associated with lower presenteeism driven by psychological strain.

Within this framework, self‐efficacy and leader–member exchange (LMX) serve as the two key cognitive filters through which nurses process leadership information. Self‐efficacy reflects nurses’ appraisal of their own competence; LMX captures their perception of supervisor support. Both are precisely the types of informational cues that SIPT highlights [9]. Nurses with greater self‐efficacy adopt proactive coping strategies when facing health challenges; those with high‐quality LMX perceive stronger organizational backing, easing the psychological pressure to work while ill [12].

Based on this theoretical foundation, we build a moderated mediation model to trace the pathway from AL to nurses’ presenteeism via self‐efficacy, with LMX moderating the first stage of this process.

AL combines the inspirational, intrinsic‐motivation‐oriented behaviors of transformational leadership with the role‐clarifying, reward‐and‐accountability structure of transactional leadership [10]. Because clinical nursing simultaneously requires high task standardization and situation‐specific judgment, neither behavioral repertoire alone is likely to be sufficient. Transformational behaviors may sustain engagement and meaning at work, whereas transactional behaviors establish clear expectations and procedural accountability; the two are theorized to operate in a complementary rather than an interchangeable manner [11]. Importantly, however, leadership behavior is a contextual rather than a curative factor: it can shape the psychosocial conditions in which nurses work, but it cannot compensate for inadequate staffing, unmanageable workloads, or the absence of sick‐leave protection.

This behavioral repertoire extends beyond information sharing and top‐down communication; it involves attention to employees’ concerns and professional development. When leaders listen and engage, subordinates may perceive greater support, which is associated with higher job satisfaction and stronger organizational commitment. Through this mechanism, a supportive work environment may ease the psychological burden on nurses, and the pressure to attend work while unwell may be correspondingly reduced. In this way, a supportive relational context may attenuate one psychosocial antecedent of presenteeism; it does not, however, remove the structural conditions that give rise to it.

Self‐efficacy refers to an individual’s confidence in their own capabilities. Research indicates that AL provides employees with support and guidance that may help them build confidence in task accomplishment and may be associated with higher self‐efficacy [13, 14]. Through open communication strategies, AL may support employees in developing higher self‐efficacy, which may in turn be associated with stronger identification with leadership behaviors and more positive mindsets and actions. Studies suggest that in challenging tasks, leaders’ exemplary behaviors are associated with employees’ internal perceptions, effort levels, and efficacy expectations, positioning leadership as a contextual factor in self‐efficacy development [14, 15]. Individuals with high self‐efficacy believe they can harness their cognitive abilities and skills to control their environment and manage unforeseen events [16]. Self‐efficacy has been repeatedly associated with lower presenteeism in previous empirical work [12, 15]. For clinical nurses specifically, self‐efficacy reflects their confidence in performing professional nursing activities, and this confidence has been linked to their psychological well‐being. Higher self‐efficacy may help nurses better address health issues and may be associated with better mental health. Compared with their low‐efficacy counterparts, nurses reporting higher self‐efficacy may be better positioned to recognize and respond to health‐related strain; however, self‐efficacy is best understood as a psychological resource that operates within, and is constrained by, the organizational conditions described above.

LMX captures the quality of the reciprocal relationship between supervisors and subordinates. Prior research has established that LMX serves as a robust predictor of employees’ work attitudes, job satisfaction, communication effectiveness, decision‐making quality, organizational citizenship behavior, organizational commitment, and turnover intentions [17, 18].

Within the pathway from AL to self‐efficacy, LMX functions as a perceptual filter. When LMX is of high quality, nurses tend to interpret their leader’s messages and behaviors through a favorable lens; they are receptive to guidance and open to support, which allows leadership inputs to be associated with enhanced work confidence and sounder decision‐making. When LMX is low, however, nurses may be more prone to view the same leadership cues with suspicion or resistance. Beyond routine task‐related exchanges, they may find it difficult to access genuine support, and the resulting erosion of trust may weaken the positive association that AL might otherwise have with their self‐confidence.

To sum up, the existing literature has separately documented the pairwise relationships among AL, self‐efficacy, LMX, and nurses’ presenteeism. Yet how these four factors operate in concert (and the precise mechanisms that link them) remains largely unexplored. The present study, therefore, aims to integrate these elements into a unified framework, examining their interconnections and underlying dynamics. The findings are expected to offer practical guidance for nursing administrators in designing targeted strategies and policies to address presenteeism. Drawing on the literature reviewed above, we propose the following hypotheses; the conceptual model is presented in Figure 1.

 H1: AL is negatively associated with nurses’ presenteeism.
 H2: Self‐efficacy mediates the relationship between AL and nurses’ presenteeism.
 H3: LMX moderates the relationship between AL and self‐efficacy.

**FIGURE 1:**
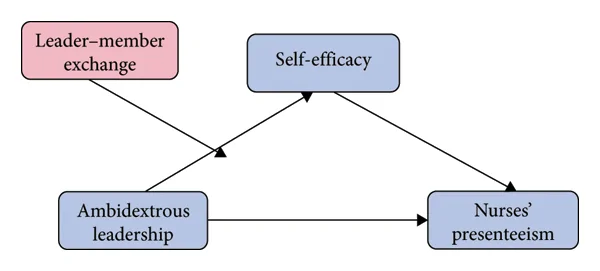
Hypothesized conceptual model. *Note*: This figure displays the conceptual framework of our study, in which ambidextrous leadership acts as the independent variable, self‐efficacy functions as the mediating variable, nurses’ presenteeism is the dependent variable, and leader–member exchange serves as the moderating variable.

## 2. Materials and Methods

### 2.1. Study Design

A cross‐sectional survey was conducted between December 2024 and January 2025, using an electronic questionnaire distributed online. The study design and execution followed the Strengthening the Reporting of Observational Studies in Epidemiology (STROBE) guidelines.

### 2.2. Participants

Owing to time and resource constraints, our team employed a convenience sampling approach to survey nurses from three Grade A tertiary hospitals in Guangzhou, Guangdong Province. These hospitals were selected for two primary reasons. First, as top‐tier healthcare institutions in China, they have large nursing workforces and well‐established management systems, offering a representative setting for examining leadership dynamics. Second, the three hospitals are comparable in size, bed capacity, and organizational structure, which may help reduce between‐site variability. Their location in Guangzhou, a major metropolitan area, also ensured good accessibility and participant willingness, further supporting the feasibility of the survey. Taken together, these considerations were intended to safeguard research validity while optimizing internal validity.

Sample size estimation was performed using G^∗^Power software (Version 3.1). Following Ferguson’s [19] criteria for social science research, we set the effect size (*f* ^2^) at 0.15 (corresponding to a small‐to‐medium effect for multiple regression analysis), statistical power (1−β) at 0.80, and significance level (*α*) at 0.05, with up to 11 predictors anticipated in the full regression model. The 11 predictors entered into the calculation were (1) AL; (2) self‐efficacy; (3) LMX; (4) the AL × LMX interaction term; and seven demographic covariates selected a priori on the basis of their documented associations with presenteeism: (5) age, (6) gender, (7) educational level, (8) years of clinical work experience, (9) marital status, (10) professional title, and (11) employment type. Based on these parameters, the minimum required sample size was 123 participants. Because the principal hypotheses concern conditional (moderated) effects, however, a larger sample was required in practice: the power to detect an interaction term is substantially lower than the power to detect a main effect of comparable magnitude, and moderated mediation estimation requires stable bootstrap resampling of the conditional indirect effect, which is sensitive to sampling error in the interaction term [20, 21]. We, therefore, raised the minimum required sample size to 200 in order to preserve adequate power for the moderation and moderated mediation analyses and to support the planned confirmatory factor analysis, for which a sample of at least 200 is commonly recommended. The figure of 200 thus represents a minimum required sample size for the focal analyses rather than a recruitment ceiling.

Inclusion criteria were (a) holding a valid nursing license, (b) at least one year of clinical work experience, and (c) informed of the study content and providing voluntary consent. Exclusion criteria included (a) nurses on external training or further education, (b) those on extended leave (more than 3 months, e.g., sick or maternity leave), and (c) nurses in standardized training or internship programs. Nurses with less than one year of experience were excluded because they remain in an orientation period, during which their perceptions of and responses to leadership may differ from those of fully integrated staff. Those on long‐term leave or external training were also excluded, as they were not actively engaged in the regular work environment during the study period.

In line with this minimum requirement, a total of 432 nurses were invited to participate, of whom 412 submitted a questionnaire (response rate = 95.4%, 412/432). Because 200 was specified as the minimum required sample size for the focal conditional‐effect analyses rather than a recruitment ceiling, distribution continued until the planned data‐collection window closed. Of the 412 submitted questionnaires, 18 were excluded, yielding 394 valid questionnaires (valid response rate = 95.6%, 394/412). Exclusions comprised questionnaires completed in less than 120 s, questionnaires with identical responses across all items, and questionnaires that failed the device/IP de‐duplication check. All exclusion decisions were made before hypothesis testing and according to prespecified criteria. Although the final sample exceeds the minimum required size, this reflects the sampling strategy described above rather than a targeted recruitment of additional participants; we acknowledge that the additional data collection may have imposed an avoidable burden on participants. See Figure 2 for details.

**FIGURE 2:**
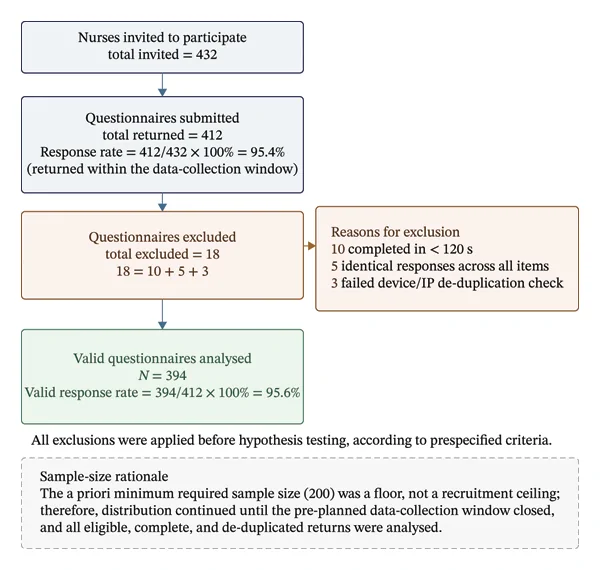
Flow diagram of participant recruitment. *Note*: Values are actual recruitment figures. Recruitment was closed at the end of the preplanned data‐collection window; all eligible, complete, and deduplicated questionnaires returned within that window were included in the analysis.

### 2.3. Instruments

#### 2.3.1. Demographic Characteristics

A self‐designed information sheet was used to collect participants’ demographic data, including age, gender, educational background, marital status, number of children, years of work experience, professional title, department, and employment type.

#### 2.3.2. AL Scale

AL was assessed using a combined scale comprising transformational and transactional leadership subscales. Transformational leadership was measured with the 8‐item scale developed by Chen et al. [22] for the Chinese context, covering charisma (4 items) and inspirational motivation (4 items). Transactional leadership was assessed using 6 items from the Multifactor Leadership Questionnaire developed by Bass et al. [23], including contingent reward (3 items) and management by exception (3 items). All items were rated on a 5‐point Likert scale from 1 (*Strongly Disagree*) to 5 (*Strongly Agree*).

In this study, AL was conceptualized as a second‐order construct, with transformational and transactional leaderships as two distinct yet complementary dimensions. This conceptualization draws on the theoretical premise that the integration of these two seemingly opposing behavioral modes is theorized to yield synergistic effects that exceed their independent contributions, enabling leaders to navigate the complex and often contradictory demands of healthcare management [11]. Rather than treating transformational and transactional leadership as mutually exclusive alternatives, this perspective emphasizes their dynamic coexistence and contextual flexibility.

It should be noted that the two subscales originate from different established instruments. The decision to combine them into a single AL construct was deliberate and grounded in both theoretical and empirical justifications. Theoretically, the integration of transformational and transactional leaderships into a unified construct is well established within the AL framework [10, 11, 24], which explicitly defines AL as the flexible combination of both behavioral repertoires. This approach also mirrors the reality of nursing management, where leaders seldom rely on a single style but alternate between transformational and transactional behaviors depending on situational demands [25]. Empirically, the two subscales demonstrated a significant positive correlation in our sample (^∗^r^∗^ = 0.62, *p* < 0.01), supporting their convergence toward a higher‐order factor. Both subscales also exhibited satisfactory internal consistency (Cronbach’s *α* = 0.87 and 0.80, respectively). In addition, the combined use of these two subscales to operationalize AL has been widely adopted in prior published studies [10, 11, 24], providing further precedent for this approach.

Based on the theoretical and empirical evidence above, the summed score of the two subscales was used as the indicator of AL in all subsequent analyses, with higher scores reflecting greater AL. The combined scale demonstrated good overall internal consistency in this study (Cronbach’s *α* = 0.89). We acknowledge that combining subscales from different sources may introduce measurement heterogeneity, and this is addressed as a limitation in the Discussion section (see Section 3.2.6).

#### 2.3.3. Stanford Presenteeism Scale‐6 (SPS‐6)

Presenteeism was measured using SPS‐6, originally developed by Koopman [26] and translated into Chinese by Zhao et al. [27]. The scale assesses declines in work efficiency over the past month due to health problems (e.g., “I’m on the job but I have trouble concentrating”). All items are rated on a 5‐point Likert scale from 1 (*Strongly Disagree*) to 5 (*Strongly Agree*). Total scores range from 6 to 30, with a median score of 15 serving as the cutoff: scores ≥ 15 indicate high levels of presenteeism, while scores < 15 indicate low levels. In this study, the Cronbach’s α coefficient for the scale was 0.92.

It should be noted that presenteeism was treated as a continuous variable in all hypothesis tests. The median split (median = 15) was used in Table 1 solely for descriptive purposes to facilitate demographic comparisons and was not employed as the basis for statistical inference.

**TABLE 1:** Characteristics of the participants (*n* = 394).

Variables	*n* (%)	Nurses’ presenteeism		*t/F*	*p*
		M	SD		
Gender				3.35	< 0.001
Male	15 (3.8)	16.48	3.97		
Female	379 (96.2)	15.88	4.64		
Age (years)				5.87	< 0.001
< 25	52 (13.2)	16.48	3.97		
25 ∼ 30	58 (14.7)	15.88	4.64		
31 ∼ 35	84 (21.3)	13.49	4.52		
36 ∼ 40	89 (22.6)	14.30	4.92		
> 40	111 (28.2)	13.70	4.27		
Education				7.25	< 0.001
College and below	48 (12.2)	16.69	4.60		
Undergraduate	326 (82.7)	14.25	4.53		
Postgraduate and above	20 (5.1)	12.95	4.38		
Work experience (years)				7.92	< 0.001
0 ∼ 5	89 (22.6)	16.33	4.33		
6 ∼ 10	54 (13.7)	14.63	4.20		
11 ∼ 15	105 (26.6)	13.24	4.57		
> 15	146 (37.1)	14.19	4.62		
Marital status				8.44	< 0.001
Unmarried	128 (32.5)	15.81	4.34		
Married	262 (66.5)	13.82	4.61		
Divorced	4 (1.0)	15.25	3.95		
Number of children				10.08	< 0.001
0	142 (36.0)	15.83	4.35		
1	129 (32.7)	13.83	4.50		
≥ 2	123 (31.2)	13.60	4.68		
Department				2.55	0.014
Internal medicine	150 (38.1)	14.18	4.86		
Surgery	101 (25.6)	14.85	4.28		
Gynecology and pediatrics	20 (5.1)	14.60	3.87		
Emergency department	11 (2.8)	19.27	4.10		
ICU	26 (6.6)	13.42	4.08		
Operating room	22 (5.6)	15.59	5.22		
Outpatient department	19 (4.8)	14.00	4.74		
Others	45 (11.4)	13.69	4.19		
Professional title				4.83	0.003
Nurse	72 (18.3)	15.81	4.30		
Senior nurse	71 (18)	15.44	4.51		
Nurse in charge	180 (45.7)	13.79	4.73		
Deputy chief nurse and above	71 (18)	13.92	4.30		
Mode of employment				−2.17	0.031
On the official payroll	247 (62.7)	14.09	4.57		
Contractual employee	147 (37.3)	15.13	4.61		

*Note:* M, mean, *t*, independent‐samples *t* test; F, one‐way analysis of variance (ANOVA). *p* values are reported because this table presents exploratory comparisons of presenteeism across demographic groups.

Abbreviation: SD, standard deviation.

#### 2.3.4. General Self‐Efficacy Scale (GSES)

Self‐efficacy was assessed using the GSES, adapted for the Chinese context by Schwarzer et al. [28]. The scale has a unidimensional structure comprising 10 items, each rated on a 5‐point Likert scale from 1 (*Strongly Disagree*) to 5 (*Strongly Agree*). Total scores, therefore, range from 10 to 50, with higher scores indicating greater self‐efficacy. In this study, the Cronbach’s α coefficient for the scale was 0.74.

#### 2.3.5. LMX Scale

LMX was assessed using the scale developed by Graen and Uhl‐Bien [29], which measures the level of leadership support and trust perceived by nurses. The scale has a unidimensional structure comprising 7 items, each rated on a 5‐point Likert scale from 1 (*Strongly Disagree*) to 5 (*Strongly Agree*). Sample items include “I know how to support my leader” and “I have a good working relationship with my leader.” Total scores range from 7 to 35, with higher scores indicating higher quality of exchange between leaders and employees. In this study, the Cronbach’s α coefficient for the scale was 0.80.

### 2.4. Data Collection

Before the survey, all participants were informed of the research purpose and assured of their right to decline or withdraw at any time, with full anonymity and confidentiality guaranteed throughout the process. The cross‐sectional survey was conducted using the Wenjuanxing online questionnaire platform. The survey link was distributed through the hospital nursing department to WeChat groups of ward head nurses, who then forwarded it to nurses in their respective departments. To ensure response quality, specific completion guidelines were provided before distribution. Technical measures were also implemented to restrict submissions to one per device and IP address. Questionnaires completed in less than 120 s or those with identical answers across all items were excluded from analysis.

### 2.5. Statistical Analysis

Statistical analyses were performed using IBM SPSS Statistics (Version 27.0) and the SPSS PROCESS Macro (Version 4.2). Before constructing the interaction term, all continuous variables (AL, self‐efficacy, and LMX) were mean (M)‐centered to reduce multicollinearity between the interaction term and its constituent variables, as recommended by Aiken and West [30].

Descriptive analyses were first conducted, with continuous variables presented as M and standard deviations (SDs) and categorical variables as frequencies and percentages. One‐way ANOVA and independent‐sample *t*‐tests were used to examine differences in nurses’ presenteeism across demographic characteristics. Pearson correlation analysis was then employed to evaluate the bivariate relationships among the main variables.

To test the mediating role of self‐efficacy between AL and nurses’ presenteeism, we applied PROCESS Model 4. Model 7 was used to examine the moderating effect of LMX on the relationship between AL and self‐efficacy. To further clarify the nature of this moderation, a simple slope test was conducted to assess the association between AL and self‐efficacy at high and low levels of LMX.

The significance of the indirect effect was assessed using a bootstrap procedure with 5000 resamples. A 95% confidence interval (CI) that did not include zero was considered indicative of a significant effect. All statistical tests were two‐tailed, and *p* values below 0.05 were deemed statistically significant.

## 3. Results and Discussion

### 3.1. Results

#### 3.1.1. Common Method Bias Test

Given that all questionnaires were self‐reported by participants, we conducted Harman’s single‐factor test on all variable items to assess the potential influence of common method bias. The results showed that seven factors had eigenvalues exceeding 1. Before rotation, the first principal component accounted for 26.0% of the total variance, which fell below the critical threshold of 40%. This suggests that common method bias did not pose a serious threat to the validity of our findings.

#### 3.1.2. Demographic Characteristics of Participants

One‐way analyses were conducted with general demographic characteristics as independent variables and nurses’ presenteeism scores as the dependent variable. The results revealed statistically significant differences in presenteeism scores across groups categorized by age, gender, education level, years of work experience, marital status, number of children, department, professional title, and employment type (*p* < 0.05). Detailed results are presented in Table 1.

#### 3.1.3. Correlation Analysis

Pearson correlation analysis revealed that AL was positively correlated with self‐efficacy (*r* = 0.442, *p* < 0.01) and negatively correlated with presenteeism (*r* = −0.282, *p* < 0.01). Self‐efficacy was also negatively correlated with presenteeism (*r* = −0.278, *p* < 0.01). The complete correlation matrix is presented in Table 2.

**TABLE 2:** Correlations among study variables (*n* = 394).

	M	SD	AL	Self‐efficacy	LMX	Presenteeism
AL	56.80	6.33	1			
Self‐efficacy	37.12	5.08	0.442^∗∗^	1		
LMX	28.27	3.46	0.564^∗∗^	0.442^∗∗^	1	
Presenteeism	14.48	4.61	−0.282^∗∗^	−0.278^∗∗^	−0.479^∗∗^	1

*Note:* M, mean; LMX, leader–member exchange.

Abbreviations: AL, ambidextrous leadership; SD, standard deviation.

^∗∗^ *p* < 0.01.

#### 3.1.4. Mediating Effects of Self‐Efficacy

PROCESS Model 4 was used to examine the mediating role of self‐efficacy in the relationship between AL and nurses’ presenteeism. The results showed that AL significantly and positively predicted self‐efficacy (*β* = 0.421, *p* < 0.001) and significantly and negatively predicted presenteeism (*β* = −0.243, *p* < 0.001). When self‐efficacy was entered as a mediator, it remained a significant negative predictor of presenteeism (*β* = −0.191, *p* < 0.001), while the direct predictive effect of AL on presenteeism also remained significant (*β* = −0.163, *p* = 0.003) (Table 3).

**TABLE 3:** Mediating effect of self‐efficacy in the association between ambidextrous leadership and nurses’ presenteeism.

Independent variable	Model 1 (nurses’ presenteeism)		Model 2 (self‐efficacy)		Model 3 (nurses’ presenteeism)	
	*β*	*p*	*β*	*p*	*β*	*p*
AL	−0.243	< 0.001	0.421	< 0.001	−0.163	0.003
Self‐efficacy	—	—	—	—	−0.191	< 0.001
*R* ^2^	0.144		0.230		0.172	
F	65.94^∗∗∗^		117.09^∗∗∗^		40.61^∗∗∗^	

*Note:* —, not applicable. *F* values were recomputed from the reported *R* ^2^ with *N* = 394 and the number of predictors in each model (Model 1: *k* = 1; Model 2: *k* = 1; Model 3: *k* = 2), following *F* = (*R* ^2^/*k*)/[(1−*R* ^2^)/(N−k−1)].

Abbreviation: AL, ambidextrous leadership.

^∗∗∗^ *p* < 0.001.

Bootstrap analysis indicated that self‐efficacy partially mediated the relationship between ambidextrous leadership and nurses’ presenteeism. The indirect (mediating) effect was −0.059 (unstandardized coefficient), with a 95% CI of [−0.108, −0.023]. The mediating effect accounted for 33.3% of the total effect (calculated based on unstandardized coefficients) (Table 4).

**TABLE 4:** Total, direct, and indirect effects of the mediation model.

	Effect (b)	Proportion (%)	95% CI
Total effect	−0.177	100	[−0.248, −0.106]
Direct effect	−0.118	66.7	[−0.195, −0.042]
Indirect effect	−0.059	33.3	[−0.108, −0.023]

*Note:* Effects are unstandardized coefficients (b); the proportion is expressed as a percentage of the total effect.

Abbreviation: CI, confidence interval.

#### 3.1.5. Moderating Effects of LMX

PROCESS Model 7 was employed to examine the moderating role of LMX. The results showed that AL significantly and positively predicted self‐efficacy (*β* = 0.216, *t* = 5.08) and significantly and negatively predicted nurses’ presenteeism (*β* = −0.118, *t* = −3.03). Self‐efficacy also significantly and negatively predicted presenteeism (*β* = −0.173, *t* = −3.61). The interaction term between AL and LMX significantly and positively predicted self‐efficacy (*β* = 0.043, *t* = 3.77), indicating that LMX positively moderated the relationship between AL and self‐efficacy (Table 5). The regression models accounted for 30.8% of the variance in self‐efficacy (Model 1) and 41.5% of the variance in nurses’ presenteeism (Model 2), with both models reaching statistical significance (*F* = 14.13, *p* < 0.001; *F* = 24.64, *p* < 0.001, respectively).

**TABLE 5:** Moderating effect of LMX in the first stage of the mediation model (PROCESS Model 7).

Variables	Model 1 (self‐efficacy)			Model 2 (presenteeism)		
	*β*	SE	*t*	Β	SE	*t*
Age	−0.286	0.472	−0.60	−0.664	0.467	−1.42
Gender	−1.325	1.147	−1.16	−3.509	1.136	−3.09^∗∗∗^
Education	1.138	0.619	1.84	−1.018	0.615	−1.66
Work experience	−0.359	0.569	−0.63	0.331	0.561	0.59
Marital status	1.255	0.777	1.62	0.007	0.769	0.01
Number of children	−0.367	0.417	−0.88	−0.824	0.413	−1.99^∗^
Department	−0.043	0.089	−0.49	−0.028	0.088	−0.31
Professional title	0.962	0.432	2.22^∗^	0.530	0.430	1.23
Mode of employment	0.841	0.592	1.42	−0.161	0.585	−0.27
AL	0.216	0.043	5.08^∗∗∗^	−0.118	0.039	−3.03^∗∗^
Self‐efficacy	—	—	—	−0.173	0.048	−3.61^∗∗∗^
LMX	0.376	0.077	4.91^∗∗∗^	—	—	—
AL × LMX	0.043	0.011	3.77^∗∗∗^	—	—	—
*R* ^2^	0.308			0.415		
F	14.13^∗∗∗^			24.64^∗∗∗^		

*Note:* F values were recomputed from the reported *R* ^2^ with *N* = 394 and the number of predictors in each model (Model 1: *k* = 12; Model 2: *k* = 11), following *F* = (*R* ^2^/*k*)/[(1−*R* ^2^)/(N−k−1)]. LMX, leader–member exchange.

Abbreviations: AL, ambidextrous leadership; SE, standard error.

^∗^ *p* < 0.05.

^∗∗^ *p* < 0.01.

^∗∗∗^ *p* < 0.001.

To further clarify the nature of the moderation, we conducted a simple slope test and plotted the moderated effects (Figure 3). The findings indicated that when LMX was low, the positive association between AL and self‐efficacy was attenuated; conversely, when LMX was high, this association was strengthened.

**FIGURE 3:**
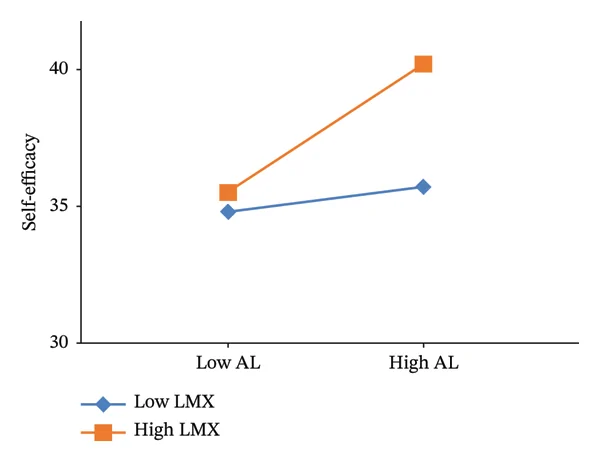
The interactive effect of leader–member exchange and ambidextrous leadership on self‐efficacy. Note. The horizontal axis represents ambidextrous leadership (low vs. high), and the vertical axis represents self‐efficacy. The two slopes compare the association between ambidextrous leadership and self‐efficacy under high and low LMX conditions. When LMX was high, self‐efficacy was higher under high ambidextrous leadership; when LMX was low, the association was weaker.

To validate the moderated mediation effect, we examined the conditional indirect effects of AL on nurses’ presenteeism via self‐efficacy at three levels of LMX: the M and one SD above and below the M. The bias‐corrected percentile bootstrap analysis revealed that the indirect effect was significant when LMX was at the M (effect = −0.037, SE = 0.016, 95% CI [−0.075, −0.014]) and at one SD above the M (effect = −0.063, SE = 0.026, 95% CI [−0.125, −0.024]) but not significant when LMX was one SD below the M (effect = −0.012, SE = 0.011, 95% CI [−0.036, 0.007]). The index of moderated mediation was −0.008 (SE = 0.004, 95% CI [−0.016, −0.002]), indicating that the mediating effect of self‐efficacy was moderated by LMX.

The two slopes compare high and low AL under different LMX conditions. When LMX was high, self‐efficacy was higher under high AL; when LMX was low, the association between AL and self‐efficacy was weaker.

Finally, the bias‐corrected percentile bootstrap analysis further demonstrated that the indirect effect of AL on nurses’ presenteeism via self‐efficacy varied across different levels of LMX. The index of moderated mediation was −0.008, with a 95% CI of [−0.016, −0.002], which did not include zero, indicating a significant moderated mediation effect (Table 6).

**TABLE 6:** Conditional indirect effects of ambidextrous leadership on nurses’ presenteeism at different levels of LMX.

LMX level	Effect (b)	SE	95% CI
M − 1 SD (low)	−0.012	0.011	[−0.036, 0.007]
M (mean)	−0.037	0.016	[−0.075, −0.014]
M + 1 SD (high)	−0.063	0.026	[−0.125, −0.024]
Index of moderated mediation	−0.008	0.004	[−0.016, −0.002]

*Note:* Conditional indirect effects were estimated with bias‐corrected percentile bootstrap (5000 resamples); an effect is significant when the 95% CI does not include zero. LMX, leader–member exchange; M, mean.

Abbreviations: CI, confidence interval; SD, standard deviation; SE, standard error.

### 3.2. Discussion

This study constructed a moderated mediation model to explore the mechanisms linking AL to nurses’ presenteeism. The results showed that AL negatively predicted presenteeism, with self‐efficacy partially mediating this relationship. LMX positively moderated the first stage of the mediation pathway: when LMX was high, the positive association between AL and self‐efficacy was stronger. These findings deepen our understanding of the underlying mechanisms and offer practical targets for intervention.

Regarding demographic characteristics, descriptive data showed that nurses with bachelor’s or higher degrees, intermediate or senior professional titles, and permanent employment status reported lower presenteeism scores. These patterns are broadly consistent with previous research suggesting that higher education and professional title may facilitate resource access and stress coping [31], while permanent employment may foster stronger organizational commitment [3]. However, we caution against overinterpreting these associations: education and title do not equate to overall competence, and the relationship between the employment type and presenteeism warrants further investigation. As these demographic factors were included primarily as control variables rather than focal predictors, we refrain from extensive interpretation and focus our discussion on the core mediation and moderation findings in the following.

#### 3.2.1. Presenteeism Across Demographic Characteristics

##### 3.2.1.1. AL and Nurses’ Presenteeism

Our finding of a negative correlation between AL and presenteeism aligns with prior research [32], suggesting that AL is associated with fewer negative work behaviors stemming from nurses’ health problems. Given the persistently high prevalence of presenteeism among Chinese nurses [5], the probability of clinical errors may rise accordingly, which may compromise care quality and may in turn be associated with productivity loss and increased organizational costs (including direct economic losses and expenditures on time and human resource management) [33]. Deficiencies in human resource management and shifts in the organizational environment both contribute to nurse presenteeism. AL is theorized to integrate two seemingly opposing styles and may thereby yield synergistic benefits to resolve managerial tensions [25]. Transformational leadership is associated with emotional support through vision‐based motivation and individualized care, which may contribute to satisfaction and work meaningfulness [34]; transactional leadership is associated with task performance and responsibility through reward‐punishment mechanisms [35]. That said, over‐idealized transformational leadership that disregards nurses’ actual capabilities may become a source of stress, while transactional leadership that leans too heavily on extrinsic incentives without emotional engagement may induce anxiety. Either extreme may be associated with adverse outcomes. Organizational leaders may consider cultivating a balanced approach that draws on the strengths of both styles, which may shape nurses’ behavioral attitudes at both emotional and task levels and may be associated with lower presenteeism.

#### 3.2.2. Mediating Role of Self‐Efficacy

Self‐efficacy partially mediated the relationship between AL and nurses’ presenteeism, indicating that AL was associated with higher self‐efficacy, which was in turn associated with lower presenteeism—a result consistent with previous work [36]. Drawing on social information processing theory, AL may flexibly address managerial conflicts through its dual behavioral features and may foster a supportive and open environment. On one hand, granting nurses greater decision‐making authority and autonomy may strengthen their work confidence; when their capabilities are recognized, nurses may be more inclined to take proactive approaches. On the other hand, emotional support may contribute to more positive self‐appraisals and a more stable sense of professional confidence. Nurses with higher self‐efficacy may be better able to recognize their own negative emotional states and may be more willing to proactively communicate with leaders or colleagues to seek timely solutions, which may in turn be associated with lower presenteeism. Taken together, these patterns suggest that self‐efficacy may represent one modifiable target, alongside the structural measures described above; managers may consider tiered competency‐based training programs, which are progressive strategies that help nurses clearly understand goals and improvement areas, with each achievement potentially reinforcing their efficacy beliefs.

#### 3.2.3. Moderating Role of LMX

Finally, LMX positively moderated the relationship between AL and self‐efficacy: the higher the LMX quality, the stronger the association between AL and self‐efficacy. Under high‐quality LMX, a closer working relationship develops between leaders and nurses, in which nurses more readily interpret leadership cues and gain access to greater work resources (e.g., training opportunities and information sharing) and emotional support [24]. These resources may support psychological resilience and efficacy beliefs when facing difficulties. In return, nurses proactively upgrade their skills to handle various emergencies, which may in turn be associated with lower presenteeism [37]. Conversely, under low‐quality LMX, nurses may perceive a more peripheral relationship and interpret the same leadership behaviors as differential treatment, which may engender defensiveness and resistance and weaken self‐efficacy; in turn, they may disengage from collaborative effort, which may reduce team effectiveness [38]. Managers may consider allocating resources fairly and adopting transparent practices to reduce perceptions of favoritism. For nurses with low LMX, team‐based tasks may be designed to facilitate leader–member interaction and gradually rebuild trust. Regular psychological assessment and targeted resource support may help stabilize team morale and support the development of both individuals and the organization.

#### 3.2.4. Ethical Boundaries and Potential Managerial Misuse

The findings of this study should be read within clear ethical boundaries. Presenteeism is a consequence of organizational and occupational‐health conditions, and the measures that most directly address it are structural: reasonable scheduling, adequate sick‐leave protection, workload adjustment, sufficient staffing, and accessible occupational health services. These are obligations of nursing management and healthcare institutions, grounded in nurses’ rights to health and safe working conditions, and they are not conditional on the psychological attributes of individual nurses. Self‐efficacy, as examined here, must, therefore, be understood as a complementary rather than a substitutive resource. It is a relational and modifiable mechanism that may support nurses’ capacity to navigate health‐related strain when structural conditions are, otherwise, adequate; it is not a remedy for structural deficiency. We explicitly caution against two managerial misreadings of our findings. The first is the substitution error: treating self‐efficacy training, resilience programs, or psychological support as an acceptable alternative to staffing, scheduling, and sick‐leave reform. Where such structural deficiencies persist, individual‐level intervention risks relocating responsibility from the organization to the nurse and may reproduce the very expectation—that nurses should remain productive while unwell—that presenteeism research seeks to challenge. The second is the measurement error: using self‐efficacy scores as a performance indicator, which would convert a supportive construct into an instrument of appraisal and could discourage nurses from disclosing health problems. We accordingly frame the practical value of our findings as conditional. Within institutions that already provide adequate structural protection, attention to leader–nurse relational quality and to nurses’ confidence in managing work demands may add incremental benefit. In the absence of such protection, leadership and psychological intervention should not be presented, or relied upon, as a substitute for organizational accountability. Future research should examine whether the relational pathway identified here operates differently under varying levels of structural support and should incorporate organizational‐level measures such as staffing ratios, sick‐leave policy, and workload intensity so that the relative contribution of structural and psychological factors can be assessed directly. We also regard the absence of such organizational measures as a limitation of the present study (see Section 3.2.7).

#### 3.2.5. Practical Implications

The practical implications of this study are best understood as two tiers of managerial action, ordered by priority. The first tier comprises organizational and structural responsibility, which remains primary. Reducing presenteeism among nurses requires reasonable and predictable scheduling, workload adjustment where demands exceed capacity, staffing levels adequate to the clinical workload, genuine access to sick leave without penalty or stigma, and the provision of occupational health services. These measures protect nurses’ health and rights directly and are necessary conditions for patient safety. Where they are absent, no leadership or psychological intervention can be expected to compensate. The second tier comprises relational and psychological measures, which are complementary. Our findings suggest that AL, the contextual balancing of transformational and transactional behaviors, was associated with higher nurse self‐efficacy and that self‐efficacy was in turn associated with lower presenteeism. The association was stronger where LMX was of higher quality. Managers may, therefore, consider leader development that helps nursing leaders adjust their behavioral repertoire to situational demands and attention to the quality and equity of leader–nurse relationships, including transparent communication and equitable allocation of resources and opportunities across the whole team rather than a subset of members [38]. For nurses reporting lower LMX, structured interaction, such as team‐based task design and regular one‐to‐one contact, may help rebuild trust. These two tiers are not interchangeable. Relational and psychological measures should be positioned as complements to structural provision, not as substitutes for it, and should not be deployed to justify expectations that nurses work while unwell (see Section 3.2.4). In settings where structural conditions are inadequate, priority should be given to correcting them before, or at least alongside, implementing individual‐level interventions.

#### 3.2.6. Limitations and Suggestions for Future Study

Several limitations of this study should be acknowledged.

First, the cross‐sectional design precludes causal inferences among the variables. Future research would benefit from longitudinal designs to track changes in nurses’ presenteeism over time and to better establish causal directions.

Second, reliance on self‐reported data, without objective measures, inevitably introduces reporting and recall biases. Incorporating objective assessment tools or qualitative approaches in future studies could improve the reliability and interpretability of the findings.

Third, convenience sampling from three tertiary hospitals in Guangzhou limits the generalizability of our conclusions. Our sample consisted predominantly of nurses from large urban hospitals with well‐structured management systems and higher staffing ratios, which differ considerably from secondary or primary facilities in terms of organizational support and leadership contexts. The observed relationships may not directly transfer to nurses in smaller or rural settings. Moreover, Guangzhou, as a developed metropolitan city, may not fully represent the broader national landscape, where regional variations could influence leadership practices and outcomes. Future studies should include more diverse samples across multiple provinces and hospital tiers to strengthen external validity.

Fourth, we operationalized AL as a second‐order construct by combining two subscales from different sources. Although we provided both theoretical and statistical justifications for this approach, such a combination may introduce measurement heterogeneity. Future efforts could focus on developing a unified AL scale specifically tailored to the healthcare context, which would offer a more standardized and precise measurement tool.

## 4. Conclusions

The findings support the hypothesized moderated mediation model in which self‐efficacy partially mediates the association between AL and nurses’ presenteeism, and in which LMX moderates the first stage of that pathway. As the design was cross‐sectional, these associations cannot establish causal direction and should be interpreted as consistent with, rather than confirmatory of, the proposed model.

First, the primary responsibility for reducing presenteeism rests with organizational and structural provision: reasonable scheduling, workload adjustment, adequate staffing, sick‐leave protection, and access to occupational health services. These measures address the conditions that produce presenteeism and should take precedence over individual‐level intervention.

Second, self‐efficacy may represent one complementary and modifiable target. Where structural support is already adequate, interventions that strengthen nurses’ confidence in managing work demands, such as tiered competency‐based training with regular constructive feedback and shared decision‐making, may add incremental benefit. Such interventions are complementary to, and cannot substitute for, organizational accountability and should not be used to place responsibility for working while unwell on individual nurses.

Third, the moderating role of LMX points to the importance of equitable leader–member relationships. Managers may consider transparent communication and fair allocation of resources across the entire team rather than concentrating attention on a subset of members. For those reporting lower LMX, proactive outreach, such as one‐to‐one meetings, may help rebuild trust and may support more consistent access to leadership support across the team.

In summary, the findings support the hypothesized model in which self‐efficacy partially mediated the association between AL and nurses’ presenteeism and in which LMX conditioned the first stage of that pathway. Because the design was cross‐sectional, these associations are correlational and cannot establish causal direction. Structural provision (reasonable scheduling, workload adjustment, adequate staffing, sick‐leave protection, and occupational health support) remains the primary responsibility of nursing management; leadership development and attention to nurses’ self‐efficacy may represent complementary measures that support, but cannot replace or substitute for, that responsibility.

**Nomenclature**
AL: Ambidextrous leadership
ANOVA: Analysis of variance
CI: Confidence interval
GSES: General Self‐Efficacy Scale
LMX: Leader–member exchange
M: Mean
SD: Standard deviation
SE: Standard error
SPS‐6: Stanford Presenteeism Scale‐6

## Author Contributions

Study design: Xiuran Zhou; data collection: Ying Yu; data analysis: Xiuran Zhou; study supervision: Xiaoying Yan; manuscript writing: Xiuran Zhou, Shifang Zou, and Hui Peng; critical revisions for important intellectual content: Xiuran Zhou.

## Funding

The authors declare that no financial support was received for the research and/or publication of this article.

## Ethics Statement

The Ethics Committee of Guangzhou First People’s Hospital approved this study (Approval No.: K‐2024‐149‐01). All research methods adhered to relevant guidelines and regulations. Every participant provided written informed consent for their involvement in this study. The research strictly followed the ethical standards set by the committee overseeing human experimentation and was in line with the Helsinki Declaration.

## Consent

Please see the Ethics Statement.

## Conflicts of Interest

The authors declare no conflicts of interest.

## Supporting Information

Additional supporting information can be found online in the Supporting Information section.

## Supporting information


**Supporting Information** STROBE checklist cross‐sectional.

## Data Availability

The datasets used and analyzed during the current study are available from the corresponding author upon reasonable request.
